# Guideline-based learning for standard plane extraction in 3-D echocardiography

**DOI:** 10.1117/1.JMI.5.4.044503

**Published:** 2018-11-20

**Authors:** Peifei Zhu, Zisheng Li

**Affiliations:** Hitachi, Ltd., Research and Development Group, Tokyo, Japan

**Keywords:** cardiac standard plane, echocardiography, guideline, Hough forest, coarse-to-fine, regression forest

## Abstract

The extraction of six standard planes in 3-D cardiac ultrasound plays an important role in clinical examination to analyze cardiac function. A guideline-based learning method for efficient and accurate standard plane extraction is proposed. A cardiac ultrasound guideline determines appropriate operation steps for clinical examinations. The idea of guideline-based learning is incorporating machine learning approaches into each stage of the guideline. First, Hough forest with hierarchical search is applied for 3-D feature point detection. Second, initial planes are determined using anatomical regularities according to the guideline. Finally, a regression forest integrated with constraints of plane regularities is applied for refining each plane. The proposed method was evaluated on a 3-D cardiac ultrasound dataset and a synthetic dataset. Compared with other plane extraction methods, it demonstrated an improved accuracy with a significantly faster running time of 0.8  s/volume. Furthermore, it showed the proposed method was robust for a range abnormalities and image qualities, which would be seen in clinical practice.

## 1 Introduction

Echocardiography is a necessary tool to evaluate the structure and function of the heart and associated vessels. It is a fast, easy, and noninvasive evaluation that uses ultrasound waves to produce images of the heart.^1^ Recently, echocardiography technology has continued evolving, with one of the major developments being real-time three-dimensional (3-D) echocardiography (3DE). 3DE provides a 3-D visualization of the heart, avoiding problems of 2DE, such as foreshortening, out-of-plane motion, and the need of geometric assumptions for volume estimation.

In a routine cardiac examination, clinicians usually use six standard planes, apical four chamber (A4C), apical two chamber (A2C), apical three chamber (A3C), parasternal short-axis mitral valve (PSX MV), parasternal short-axis papillary muscle (PSX PM), and parasternal short-axis apex (PSX AP), to evaluate the structure and function of the heart.^2^^,^^3^ However, in a 2-D conventional examination, the standard planes are searched by clinicians manually, which causes inefficiency problems, such as user dependency, complex operational procedures, and time consumption. With the emergence of 3DE, automatic extraction of standard planes from cardiac volume becomes possible. Therefore, developing an efficient and robust method for automatic plane extraction in 3DE is extremely important in improving the cardiac examination workflow.

Previous works have proposed automatic extraction in 3DE. Recently, deep learning-based method has become more and more popular in medical image analysis.^4^^–^^6^ In Ref. 7, a method based on fully convolutional network and multitask learning is proposed for detecting canonical reference space in 3-D fetal brain ultrasound. In another work,^8^^,^^9^ deep convolutional neural network and recurrent neural network are used to localize standard plane in fetal ultrasound. Although such methods could obtain acceptable accuracy, the complex computation and time-consuming become serious problems, especially when dealing with 3-D data (usually takes more than 1 min to acquire one plane from a volume^8^). However, according to the opinion of clinical experts, cardiac standard plane extraction is expected to be processed in less than 1 s. The results are expected to be appeared in the screen at the same time clinicians press the automatic button, so that the waste waiting time could be significantly shortened. Therefore, efficient methods are necessary for this work. Additionally, deep learning-based methods usually need to be applied on advanced graphics processing unit (GPU), and installing advanced GPU on echocardiography will also increase the cost.

On the other hand, methods based on other machine learning techniques have also been proposed. In Ref. 10, a database-driven knowledge-based approach is proposed for plane extraction. The method extracts image features from each standard plane and creates a probabilistic model.^11^ During searching, a series of detectors are applied to estimate plane parameters, i.e., translation, orientation, and scale. False hypotheses at the earlier stages are removed, while lateral hypotheses are propagated to the final stage. However, large computational complexity for obtaining all plane parameters is still a problem, and the correct plane might also be missed at an earlier stage during search. In Ref. 12, the locations of planes are considered as continuous parameters, and a regression voting approach is used to solve it. Regression forest (RF)^13^^,^^14^ incorporated with voxel class information is used to train classifiers. During testing, every voxel of the cardiac volume provides votes on the parameters of each plane. The votes from all voxels are collected to produce a probability distribution, and the location of the plane is determined by the parameter with maximum probability. However, each plane is extracted independently in this approach, which means each voxel of the volume should pass through the classifier repeatedly (six times for six standard planes). This causes large computational complexity and is time consuming. In addition, anatomical regularities of standard planes, i.e., three apical planes should pass through the same center axis (apical long axis),^2^ are not considered in Ref. 12. Such knowledge is important in diagnosis and should also be incorporated into the process of plane extraction.

**Fig. 1:**
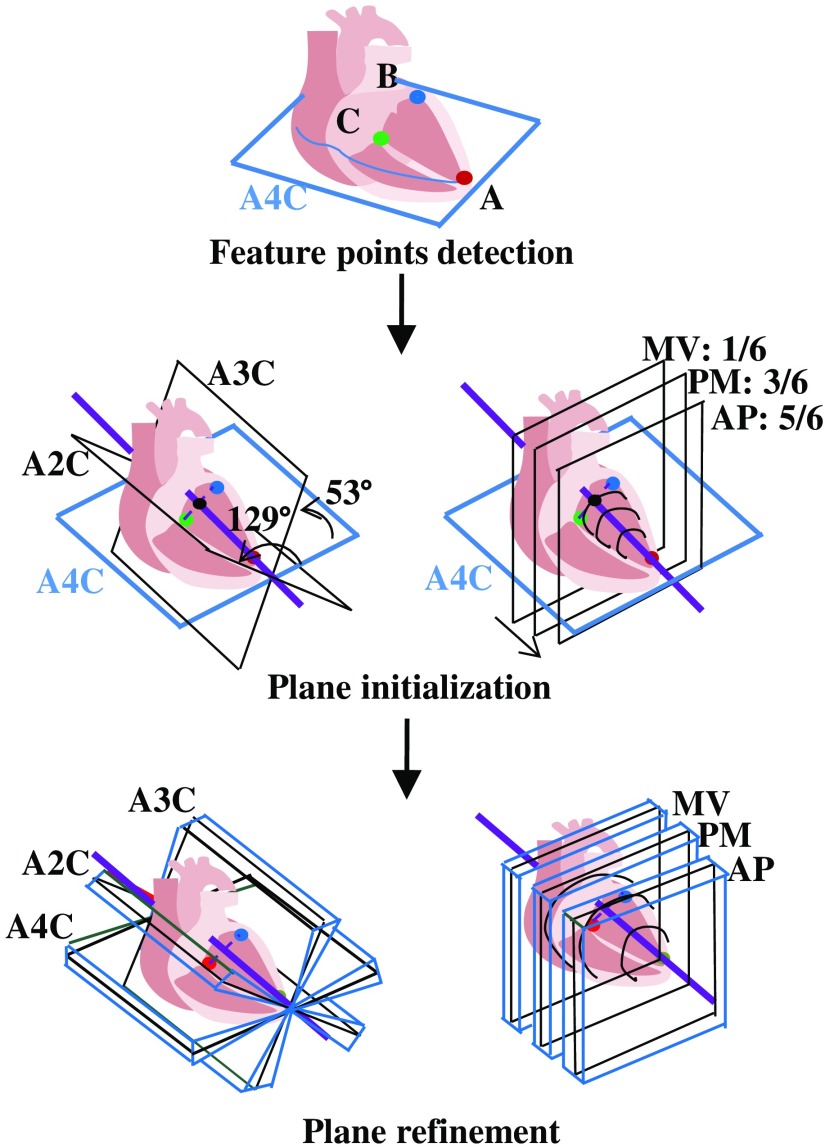
Framework of guideline-based machine learning for standard plane extraction.

This paper proposes a machine learning framework based on the cardiac ultrasound guideline (presented by the American Society of Echocardiography^2^) for standard plane extraction. The guideline has been established for clinicians to learn appropriate operation procedures for high-quality cardiac examination. The proposed method is completely based on the guideline. Each stage in the guideline is achieved using an appropriate machine learning approach that yields guideline-based machine learning. The framework of the proposed method is shown in Fig. 1, and the process is as follows.

1. Feature point detection: The guideline indicates searching the A4C plane using mitral annulus (MA) and apical features. Three anatomical feature points are selected correspondingly, and a Hough forest classifier^15^^,^^16^ with a hierarchical search is applied for detecting these points.
2. Plane initialization: The guideline indicates the anatomical regularities between A4C and the other five planes. Correspondingly, the initial locations of the other five planes are determined using these regularities.
3. Plane refinement: Refinement is needed considering individual differences around the initial location. A RF method with locations constraints is applied for plane refinement.

This work makes three main contributions. First, it presents guideline-based machine learning that incorporates machine learning approaches into each stage of the guideline. This idea can also be applied to various measurements in medical images. Second, it presents a method using a Hough forest with hierarchical search for efficiently and accurately detecting 3-D feature points. Third, location constraints are integrated into the RF for plane refinement, further improving the accuracy of plane extraction.

A preliminary version of this paper appeared in Ref. 17. The present paper contains a more detailed description of the algorithm and four additional experiments as follows. (1) A synthetic dataset with a range of abnormalities and severity is used for evaluation. It aims to show whether the proposed method is robust to heart failure samples. (2) The synthetic datasets are added with three different noise levels (0%, 10%, and 20%) to simulate different image qualities. Therefore, the influence of image quality to the accuracy of plane extraction could be evaluated. (3) Manual measurements are provided by two different observers. Interobserver variability is evaluated and compared with automatic method using two important measurements (MA diameter and left ventricle length). (4) The evaluation of the long-axis extraction is added. It aims to check whether the long-axis determined by the feature points has enough accuracy, and it further relates to the reason why the plane refinement is applied with a fixed long-axis. With the additional experiments, this work shows the proposed method is not only fast and accurate, but also robust for a wide range of data, which would be seen in clinical practice.

## 2 Standard Plane Initialization

According to the guideline,^2^ among six standard planes, the A4C plane is first extracted by using MA and apical features. In the proposed method, three anatomical feature points, including the apex, septal MA, and lateral MA, are selected correspondingly to localized plane A4C. Feature point detection is achieved using a Hough forest classifier, which is presented in Sec. 2.1. Moreover, a hierarchical search, presented in Sec. 2.2, is applied for improving the accuracy and speed. Then, as presented in Sec. 2.3, the initial locations of the six planes can be determined using the detected points and anatomical regularities all at once.

### 2.1 Hough Forest

Hough forest is used for detecting feature points. This method provides a way to map from image patches to anatomical locations. A set of random trees is learned from the training data and used to provide probabilistic votes to the target location. In this work, Hough forest is extended for 3-D point detection using 3-D image features and 3-D Hough voting. The training and testing process is described in the following subsections.

#### 2.1.1 Training process

Each tree T of Hough forest is constructed based on a set of patches $\left\{P_{i}=(I_{i},c_{i},d_{i})\right\}$, where $I_{i}$ is the appearance of the 3-D patch, $c_{i}$ is the class label that includes the positive class and negative class, and $d_{i}$ is the offset from the patch center to the object center. The proportion between object patches and background patches $C_{L}$ and the list $D_{L}=\{d_{i}\}$ of the offset vectors are stored for each leaf node L. Hough forest classifier is constructed from the root using the input patches. A key point of Hough forest is the evaluation of the binary test. The binary test is defined by a comparison of two feature values at two locations. To conduct an optimal test, the uncertainties in both the class labels and the offset vectors should decrease toward the leaves. A set of patches is defined as $A=\{P_{i}=(I_{i},c_{i},d_{i})\}$, and class label uncertainty $U_{1}(A)$ and offset uncertainty $U_{2}(A)$ are defined as follows:

$$U_{1}(A)=-|A|\cdot \sum p(c|A)\ln(p(c|A)),$$ (1)

$$U_{2}(A)=\underset{i}{\sum }{\left(d_{i}-d_{A}\right)}^{2},\;\text{when}\;c_{i}=1,$$ (2)

where |A| is the number of patches, p(c|A) is the proportion of patches with label c in set A, and $d_{A}$ is the mean offset vector over all object patches. Given a training set of patches, a pool of pixel tests $\left\{t_{k}\right\}$ is generated by randomly choosing one feature channel and two-pixel locations inside a patch. The randomized decision is made as to whether the node should minimize the class-label uncertainty or the offset uncertainty. The process can be represented as follows:

$$\arg\;\min(U_{*}(\{P_{i}|t^{k}(I_{i})=0\})+U_{*}(\{P_{i}|t^{k}(I_{i})=1\})),$$ (3)

where * is either the class label uncertainty or offset uncertainty.

#### 2.1.2 Testing process

Testing can be considered as regression and voting steps. The regression process is as follows: (1) for each voxel location p, a patch is extracted and starts regression from the root and (2) when passing each node, this patch is sorted into the left or right child node in accordance with the binary test. All pixels in the image go through the forest until they reach the leaves. During the voting process, the information stored in leaves is used to cast the probabilistic Hough votes to the location of the object center. The leaf information consists of proportion $C_{L}$ and offset vectors $D_{L}$, so $C_{L}/D_{L}$ is defined as a weight value for a vote. Each pixel in leaves carries a location p, and it votes to all locations $\left\{p-d|d\in D_{L}\right\}$ with a weight value $C_{L}/D_{L}$. After all votes from each voxel have been summed up, the 3-D Hough image can be obtained. Finally, the feature points are the locations with the maximum number of votes.

### 2.2 Hough Forest with Hierarchical Search

In Hough forest, the whole image is used during the testing step to cast the probabilistic Hough votes to the location of the object. When dealing with volume data, the regression of a huge number of 3-D patches through forest will cause massive computations. In this work, a coarse-to-fine strategy is applied to accelerate the detection process. Feature points are detected serially through a multiscale hierarchical search.

**Fig. 2:**
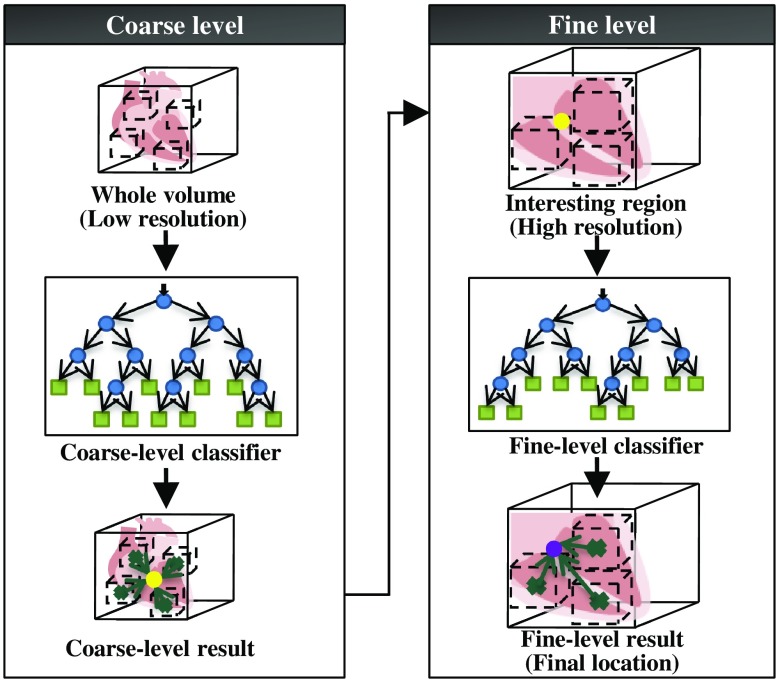
Coarse-to-fine strategy applied for the Hough forest classifier.

In the coarse-to-fine strategy, the whole image is first used to provide an estimate of the region of interest, which is then refined by using only local information. The framework is shown in Fig. 2. A coarse-level classifier and a fine-level classifier need to be trained before testing. The coarse classifier is trained using low-resolution images that are downsampled from original images. Positive patches are chosen from a bounding box region around ground-truth, and negative patches are chosen from the whole image except the positive region. The fine-level classifier is trained on a high-resolution image (original image) with a sampling region narrowed down. During the testing step, first, the input image is down-sampled, and a coarse position is localized using Hough forest coarse-level classifier. In coarse-level detection, every pixel in a low-resolution image provides a vote (Hough voting) to a potential target location. Second, in the refinement step, only pixels in the neighborhood of the coarse position are used to predict the existence of the object. By applying a coarse-to-fine strategy, the searching region has largely been cut down, so the running time is successfully shortened. Moreover, refinement searching that only uses the region closest to the target can reduce the irrelevant information and provide higher accuracy.

### 2.3 Plane Initialization Using Anatomical Regularity

The initial locations of six standard planes can be determined using three feature points and the anatomical regularity defined in Ref. 2, as shown in Fig. 1. First, the A4C plane passes through three feature points. The long-axis can also be localized by point A and the center of point B and point C. The A3C and A2C planes are intersected with the A4C plane at angles of ∼53  deg and 129 deg, respectively. Three short-axis planes (PSX MV, PM, and AP) are perpendicular to the A4C plane. According to our statistical analysis of plane location, three short-axis planes are usually localized by translating along the long-axis with proportional intervals of 1/6, 3/6, and 5/6, respectively. Therefore, the locations of six initial planes are determined.

## 3 Regression Forest for Plane Refinement

The anatomical regularities, including angles and distances in Ref. 2, are estimated on average. Because the plane location has individual differences, standard planes need to be refined. Since information close to the initial planes plays much more important roles than information that is far away, the plane refinement will be applied around their initial locations, i.e., information of atrial apex will be used to refine the A3C plane and not be used to refine the PSX AP plane. In this section, a method that incorporates location constraints into RF is proposed for plane refinement.

**Fig. 3:**
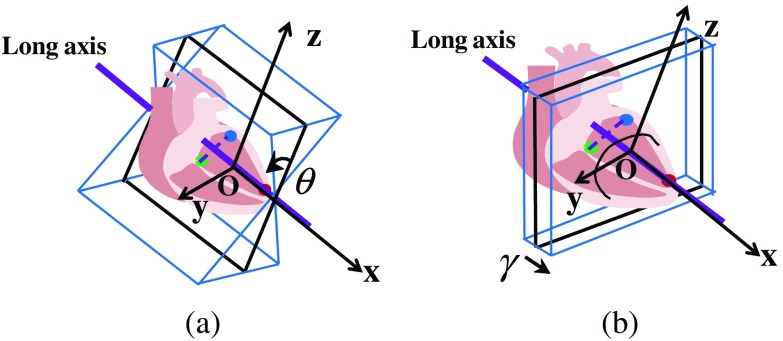
Examples of standard plane refinement: (a) Long-axis plane and (b) short-axis plane.

The refinement can be categorized into two types according to the location constraints: (1) three long-axis planes (A4C, A3C, and A2C) should pass through the long-axis and (2) three short-axis planes (MV, PM, and AP) should be perpendicular to the long-axis. Correspondingly, either angle or distance of the initial plane will be refined, as shown in Fig. 3. The reason the long-axis can be fixed is that according to the experiment result in Sec. 4.1, the long-axis direction determined by three feature points is accurate and reliable. To reduce the inference of irrelevant image information, background and object regions are also set in RF.

### 3.1 Training Process

Some important planes and parameters are first defined. The center of the 3-D volume is set as an original point O, and x, y, z coordinates are defined with the center of point O. The original plane is the xz plane, and the ground-truth plane is annotated by clinicians. In addition, the sampling plane is defined as the plane passing through the center of a sampling patch. For the long-axis planes, the sampling plane also passes through the long-axis, shown as the blue planes in Fig. 3(a), while for the short-axis planes, the sampling plane is perpendicular to the long-axis, shown as the blue planes in Fig. 3(b). An offset parameter ϕ(θ,γ) is then defined, where θ is the angle between the sampling plane and the ground-truth plane, and γ is the distance between the sampling plane and the ground-truth plane.

**Fig. 4:**
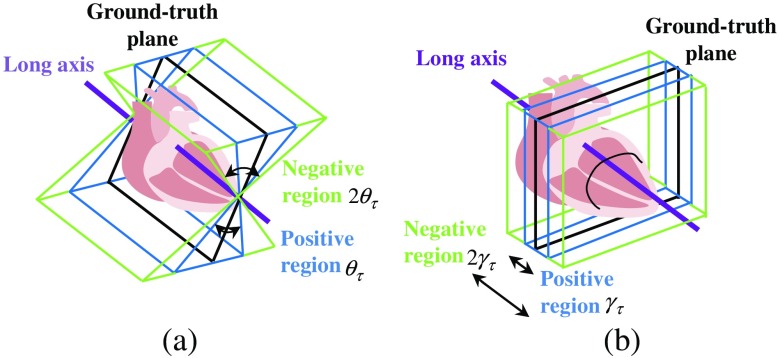
Positive region and negative region for extracting patches in training: (a) long-axis plane and (b) short-axis plane.

The training dataset comprises a set of patches $\left\{P_{i}=(I_{i},c_{i},\phi (\theta ,\gamma ))\right\}$ sampled from background and objective regions, where $I_{i}$ is the appearance of the patch and $c_{i}$ is the class label. The center of the positive patch is collected from a range with an angle less than $\theta_{\tau }\;\deg$ and a distance less than $\gamma_{\tau }$ around the ground-truth plane, as shown in Fig. 4. In practice, $\theta_{\tau }$ and $\gamma_{\tau }$ are determined as 10 deg and 5 mm, which is the maximum permissible error according to the opinion of the clinicians. The center of the negative patch is collected from a range with an angle between $\theta_{\tau }∼2\theta_{\tau }\;\deg$ and a distance $\gamma_{\tau }∼2\gamma_{\tau }$ between around the ground-truth plane. These ranges are determined by the error of the initial plane. More than 90% of the planes have an angle error of less than $2\theta_{\tau }$ and a distance error of less than $2\gamma_{\tau }$.

Each tree of the RF is constructed recursively using the input patches. Binary test is defined at each nonleaf node. During each binary test, the uncertainties in the class labels $V_{1}(A)$ and the offset angle $V_{2}(A)$ are defined as follows:

$$V_{1}(A)=-|A|\cdot \sum p(c|A)\ln(p(c|A)),$$ (4)

$$V_{2}(A)=\underset{i}{\sum }{\left(\phi_{i}-\phi_{A}\right)}^{2},\;\text{when}\;c_{i}=1,$$ (5)

where $\phi_{A}$ is the mean offset parameter over all sampled patches. For obtaining an optimal test, the node should minimize whether the class-label uncertainty or the offset parameter uncertainty, which can be represented as follows:

$$\arg\;\min(V_{*}(\{P_{i}|t^{k}(I_{i})=0\})+V_{*}(\{P_{i}|t^{k}(I_{i})=1\})),$$ (6)

where * is either class label uncertainty or offset uncertainty. Finally, for each leaf node, the proportion of the object patches and the background patches $C_{L}$, and the list $\left\{\phi_{i}\right\}$ of the offset angle are stored. Note that the offset vectors of the negative patches are not stored in the leaf.

### 3.2 Testing Process

The refinement regions are first defined. For the long-axis planes, the refinement region is set as an angle of $\left(-2\theta_{\tau },2\theta_{\tau }\right)$ around the initial planes. For the short-axis planes, the refinement region is set as a distance of $\left(-2\gamma_{\tau },2\gamma_{\tau }\right)$ centered at the initial planes. Given a unseen volume, all voxels of the refinement regions are pushed through each tree of forest until they reach leaf nodes. Leaf information consists of the proportion $C_{L}$ and the offset parameter $\left\{\phi_{i}\right\}$. The proportion can be used for determining a threshold τ to control the minimum presence of the class label at each leaf and for use as a probability for the votes this leaf generates.

**Fig. 5:**
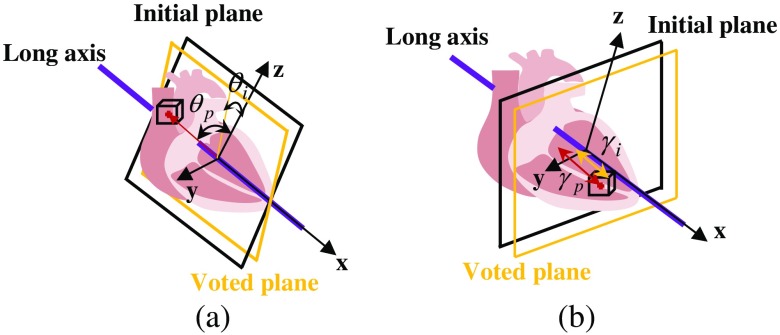
Voting the target plane using patches around the initial planes: (a) long-axis plane and (b) short-axis plane.

The voxel then votes for the location of the target plane $\phi_{t}(\theta_{t},\gamma_{t})$ using the proportion $C_{L}$ and the offset parameter $\left\{\phi_{i}\right\}$ stored in the leaf. The process is as follows. First, the plane passing through the sampling voxel and the long-axis is calculated. $\phi_{p}(\theta_{p},\gamma_{p})$ is marked as the angle and distance difference between this plane and the original plane (xz plane). Therefore, a vote on the location of the target plane $\phi_{t}=\phi_{p}-\phi_{i}$ is generated, as shown in Fig. 5. For the long-axis planes, angle component θ is used and distance component γ is equal to 0, while for the short-axis planes, distance component γ is used and angle component θ is equal to 0. All votes generated by the voxels can be summed up, and the final angle of the plane can be determined by the mean value $\overset{¯}{\phi_{t}}=\underset{L\in F}{\sum }\phi_{t}\cdot C_{L}/N$, where L∈F means all leaves in the forest, and N is the total number of votes. Finally, the target plane can determine the original plane and the voted parameter $\bar{\phi_{t}}$.

## 4 Experiments

The proposed method is evaluated on a 3-D cardiac ultrasound dataset that is available in Ref. 18 and a synthetic dataset that is available in Refs. 19 and 20.

The 3-D cardiac ultrasound dataset is a public clinical dataset, which includes 209 cardiac volumes from 15 volunteers. The data are acquired using an iE33 3-D echocardiography system (Philips Healthcare, Best, The Netherlands) with a 3-D X3-1 matrix array transducer. Full-volume acquisition mode is used in which several smaller imaging sectors are combined to form a large composite volume.^18^ All volumes are unified to a resolution of $0.5\;{\mathrm{mm}}^{3}$. Volume dimensions are around 320×347×241. Two sets of manual measurements (ground-truth) including three feature points and plane locations are provided by two observers for evaluating interobserver variation.

On the other hand, the synthetic dataset is generated by University of Leuven. It appears similar to real ultrasound recordings, yet, the myocardial motion is controlled by an E/M model in Ref. 21. By varying the parameters of the E/M model, eight sequences are generated corresponding to different pathophysiological conditions, namely: one healthy sequence; four ischemic cases, corresponding to occlusion of the proximal or distal parts of the left anterior descending coronary artery (LADprox and LADdist), of the left circumflex coronary artery (LCX) and of the right coronary artery (RCA); three simulations of dilated cardiomyopathy, of which one with a synchronous activation pattern (sync) and two dys-synchronous due to left branch bundle block (LBBBsmall and LBBBlarge). The size and the resolution of volumes are 224×176×208  voxels and $0.7\times 0.9\times 0.6\;{\mathrm{mm}}^{3}$. For each sequence, 34 volumes are included (272 volumes in total). Since a set of ground-truth of meshes is also provided, the ground-truth of the feature points and the standard planes could be easily calculated.

For improving the accuracy and evaluating the robustness of the algorithm, the following processes are also applied.

Data augmentation: To increase the variability of training data, a data augmentation scheme with artificially rotating and scaling of the original volume is applied for two datasets. A basic rotation around x-, y- and z-axis and a scaling along x-, y- and z-axis are applied, respectively. The angle (orientation) and size range of LV in the dataset are analyzed first. Cardiac LV tends to be inside the angle range of −20  deg to 20 deg around each axis and scale range of 0.8 to 1.2 times along each axis. Based on this analysis, five artificial patterns are generated with a randomly chosen angle between −20  deg to 20 deg and a randomly chosen scale between 0.8 and 1.2 times for each original volume. All generated patterns and original volumes are used in training detectors.

Cross-validation: A fivefold cross-validation scheme is applied for evaluating the clinical dataset, and an eightfold cross-validation scheme is applied for evaluating the synthetic dataset.

Generate image with different noise levels: To test the robustness of the algorithm for a range of image qualities, the synthetic data is added with three different noise levels: 0% (original image), 10%, and 20% in relative amplitude. Gaussian white noise of zero mean with 0.01 variance is used for generating noise images.

### 4.1 Evaluation of Feature Point Detection and Long-Axis Extraction

The performance of the feature point detection is first evaluated on the clinical dataset. Two methods are evaluated: (1) Hough forest and (2) Hough forest with hierarchical search (proposed method). The parameters are set as: maximum tree depth D=15, number of trees T=10, and the threshold for separating the objective leaf and background leaf τ=0.95. In addition, the image features used in this work include voxel intensity, difference of two voxel intensities, and gradient features, which are extracted by 3-D Sobel filter.^22^

Distance error is used as a metric for the evaluation. In a clinical dataset, manual points are annotated by two observers independently, and the average of the two manual measurements is used. The distance error is the Euclidean distance between the manual and the detected points. The comparison results of two methods are shown in Table 1. The distance error of each of the feature points and the mean distance error of all three points are calculated, and they are shown in mean±standard deviation format. The comparison results demonstrate that the proposed method reduces the mean distance error of the three points by about 26.1% and also improves the speed by about 10 times.

**Table 1:** Comparison of point detection between Hough forest and proposed method.

	Distance error (mm)			Run time (s)	
	Apex	Septal MA	Lateral MA	Mean	—
Hough forest	9.8±3.4	5.4±2.9	5.6±2.7	6.9±3.1	4.5
Proposed method	7.3±3.2	3.9±2.6	4.2±2.5	5.1±2.8	0.45

**Fig. 6:**
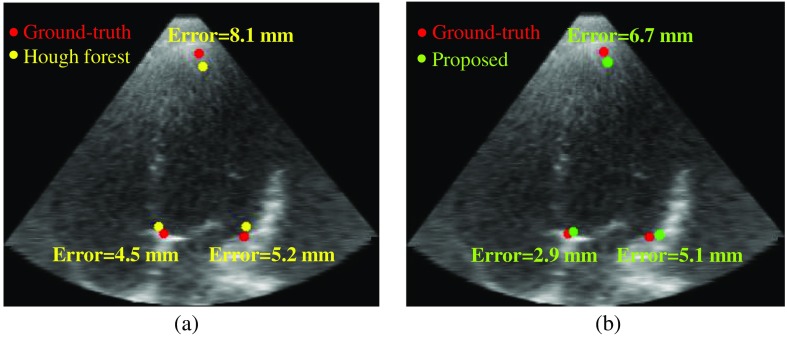
Examples of feature point detection (average-case): (a) Hough forest versus ground-truth and (b) proposed method versus ground-truth.

**Fig. 7:**
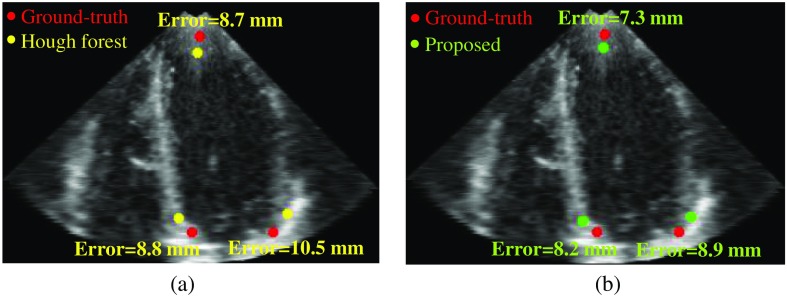
Examples of feature point detection (worst-case): (a) Hough forest versus ground-truth and (b) proposed method versus ground-truth.

Examples of detected feature points are shown in Fig. 6 (average-case) and Fig. 7 (worst case). The images are the A4C plane which is localized by three ground-truth feature points. Three detected feature points are projected to the ground-truth A4C plane. In the average-case, the apex has a larger error than the septal MA and the lateral MA because the contour above the apex is usually blurred. In the worst case, the septal MA and the lateral MA have the largest error among all samples because parts of the MA are not included in the image. However, in both cases, the proposed method shows improved accuracy for all feature points.

The evaluation result of the long-axis extraction is shown in Table 2. This experiment aims to show whether the long-axis determined by the feature points has a good accuracy. It further relates to the reason why the plane refinement is applied with a fixed long-axis. As described in Sec. 2.3, the long-axis can be localized by the apex and the center of septal MA and lateral MA. The error between the ground-truth long-axis and the extracted long-axis is calculated and is shown in Table 2. Since the ground-truth long-axis and the extracted long-axis are intersected with each other in all samples, the distance error is equal to 0. As for the angle error, the proposed method achieves a relatively small error (2.7 deg). Even though the apex is difficult to be detected, the error of the apex mainly appears in the vertical direction, which had little influence on the long-axis extraction. The horizontal location of the apex could be accurately detected since the left and right contours are usually clearly shown in the image (only one example is blurred, shown in Fig. 6). Based on this result, the long-axis is determined to be fixed during the plane refinement.

**Table 2:** Comparison of long-axis extraction between Hough forest and proposed method.

	Angle error (deg)
Hough forest^6^	3.4±1.4
Proposed method	2.7±1.2

In conclusion, the improvement in the accuracy and speed is attributed to a coarse-to-fine strategy. The searching region is largely reduced, enabling a significantly shorter running time. Moreover, only regions that are close to the target are used. This reduces the irrelevant information and provides higher accuracy.

### 4.2 Evaluation of MA Diameter

**Fig. 8:**
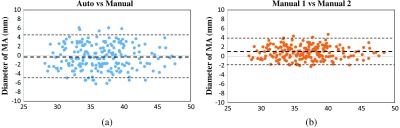
Bland–Altman plots of MA diameter measurements: (a) compare auto with manual measurements and (b) compare two manual measurements.

The diameter of the MA is important in helping the clinicians to define the etiology and mechanism of atrioventricular valve regurgitation. This item is calculated by the locations of the septal MA and lateral MA. This experiment is used in evaluating interobserver variability. Figure 8 shows the Bland–Altman plots of the MA diameter measurements. The Bland–Altman plot^23^^,^^24^ is widely used for analyzing agreement and bias between two measurements. Figure 8(a) compares the automated measurements with manual measurements on 209 cardiac volumes. Horizontal axis is the average of two manual measurements annotated by two different observers, and vertical axis is the distance error of automatic and manual measurements. This figure shows the mean difference is centered close to zero (bias=−0.27), which suggests that the automated measurement is almost unbiased to the average of two manual measurements. Additionally, there is also no bias over the hearts of different MA sizes. On the other hand, the bias between two difference observers in Fig. 8(b) is 0.79, much larger than that in (a). This indicates that individual observers may have a different opinion about the ground-truth location and be biased. As the automated method is trained with annotations from multiple observers, it naturally learns a consensus estimation across all the observers and thus less sensitive to bias.

### 4.3 Evaluation of LV Length

**Fig. 9:**
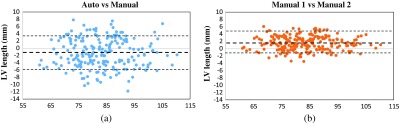
Bland–Altman plots of LV length measurements: (a) compare auto with manual measurements and (b) compare two manual measurements.

LV length is another important factor to help clinicians diagnose cardiac diseases. This item is calculated by the location of the apex and the center of septal MA and lateral MA. Figure 9 shows the Bland–Altman plots of the LV length measurements. Figure 9(a) shows when comparing the automatic results with the manual measurements, the mean difference (bias) ±1.96  SD is −1.3±4.7  mm. LV length has a relatively larger error than MA diameter because the apex is much more difficult to detect. As described in Sec. 4.2, the vertical location of the apex is especially difficult to be determined, which has a large influence on LV length measurement. On the other hand, Fig. 9(b) shows the result of comparing two manual measurements. The mean difference (bias) ±1.96  SD is 1.5±3.2  mm. The distance error also becomes larger when comparing to MA diameter. This fact shows that the apex location is usually difficult to be determined even for the clinical experts. In addition, this experiment shows that the bias of the automatic measurements is still slightly larger than the bias of two manual measurements.

### 4.4 Evaluation of Plane Extraction

Two evaluation standards are introduced, angle error and distance error, to measure the difference between the ground-truth plane and the extracted plane.^2^ The angle error between two planes is defined as the angle between the normal vector of the ground-truth plane and the normal vector of the extracted plane. The distance error between two planes is measured as the distance from an anchor on one plane to the other plane, where the anchor is the LV center. The ground-truth planes are all also annotated by two observers independently, and the average location of two measurements is used in this experiment. During the manual annotation, the standard planes are determined by image features and anatomical regularities. For example, the PSX MV plane is at the base cardiac left ventricle, and a common feature of this plane is the so-called “goldfish mouth” look of the mitral valve leaflets. In addition, a clinical criterion that determines angle error of 10 deg and distance error of 5 mm as the maximum permissible error is also used to calculate a success rate.

#### 4.4.1 Effect of plane refinement

**Fig. 10:**
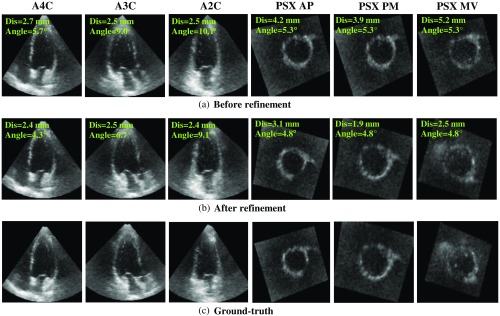
Examples of standard plane extraction (average-case). (a) Before refinement, (b) after refinement, and (c) ground-truth.

**Fig. 11:**
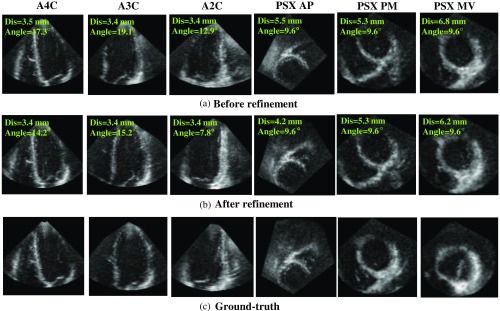
Examples of standard plane extraction (worst-case). (a) Before refinement, (b) after refinement, and (c) ground-truth.

Comparison results between applying the refinement before and after are shown in Table 3. Six standard planes are categorized into two types. The long-axis planes include A4C, A3C, and A2C, and the short-axis planes include PSX MV, PSX PM, and PSX AP. The results show that improved accuracy is achieved after refinement. The mean angle error of the long-axis planes and mean distance error of short-axis planes are reduced by 16.8% and 48.9%. In addition, the number of samples satisfied the clinical criteria (angle error less than 10 deg and distance error less than 5 mm) is increased from 127/209 (60.8%) to 168/209 (80.4%). The results demonstrate the effectiveness of the angle and distance refinement using the proposed method. Examples of standard plane extraction are shown in Fig. 10 (average-case) and Fig. 11 (worst-case). Using the refinement obviously improved detailed information such as the region near the aortic valve on the A3C plane (average-case). The worst-case is exactly the same sample as the one appeared in feature point detection. Because the detected MA have a large error, three short-axis planes have a large distance error correspondingly. The reason for this large error is that parts of the MA are not included in the image.

**Table 3:** Comparison of plane extraction between applying the refinement before and after.

	Three long-axis planes		Three short-axis planes	
	Angle (deg)	Distance (mm)	Angle (deg)	Distance (mm)
Before refinement	10.7±5.5	2.6±2.3	6.2±3.7	4.7±3.0
After refinement	8.9±5.0	2.6±2.2	6.2±3.5	2.4±2.1

#### 4.4.2 Comparison with other plane-extraction methods

The performance of the proposed method is compared to that of other plane extraction methods. The results are shown in Table 4. The average angle and the distance error of all six planes are calculated. The running time is all measured as the total extracting time of six planes, and all the experiments are run on an Intel core i7 3.6 GHz computer with 16 GB of RAM. The proposed method is compared with marginal space learning (MSL)^10^ and class-specific RF.^12^ As shown in Table 4, the angle and distance error of the proposed method is reduced by about 30% compared with those of MSL, while the running time of the proposed method is significantly shorter than that of the class-specific RF. In addition, the success rate is also calculated according to the clinical criteria (angle error less than 10 deg and distance error less than 5 mm). The proposed method achieves a success rate of 168/209 (80.4%), while MSL only has 102/209 (48.8%) and class-specific RF only has 122/209 (58.4%).

**Table 4:** Comparison results of standard plane extraction between proposed method and other plane extraction methods.

	Angle (deg)	Distance (mm)	Clinical success rate	Run time (s)
MSL^10^	11.1±7.8	3.5±2.1	102/209 (48.8%)	2
Class-specific RF^12^	6.8±4.2	4.1±3.7	122/209 (58.4%)	30
Proposed method	7.6±4.3	2.5±2.2	168/209 (80.4%)	0.8

#### 4.4.3 Evaluation on synthetic dataset with a range of abnormalities

**Fig. 12:**
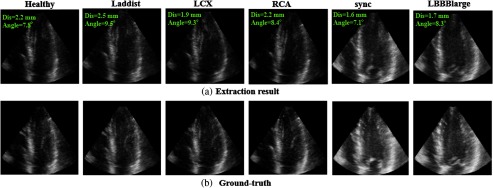
An example of standard plane extraction (synthetic dataset, original data). (a) Extraction result and (b) ground truth.

The performance of the proposed method is evaluated on the synthetic dataset (eight sequences, 272 volumes, eightfold cross-validation). This dataset includes one healthy case and seven ischemic cases. The evaluation result is shown in Table 5. There is no large difference between the healthy case and the ischemic cases. The average angle error and distance error is all smaller than the clinical maximum permissible error. An example of standard plane extraction (the A4C plane) is shown in Fig. 12. All planes including abnormal cases are correctly extracted, which suggests the proposed method is robust for a range of abnormalities.

**Table 5:** Evaluation result on synthetic dataset with a range of abnormalities.

	Three long-axis planes		Three short-axis planes	
Patient	Angle (deg)	Distance (mm)	Angle (deg)	Distance (mm)
Healthy	7.5±1.9	2.2±0.8	5.4±1.3	2.5±1.3
LADprox	9.8±2.3	2.4±0.9	5.1±1.2	2.7±1.4
LADdist	8.1±2.0	1.9±0.9	4.8±1.1	2.5±1.2
LCX	9.1±2.2	2.3±1.0	5.3±1.2	2.7±1.2
RCA	8.2±2.1	2.0±0.9	5.1±1.0	2.4±1.1
Sync	7.2±1.9	1.8±0.8	4.7±1.0	2.5±1.2
LBBBsmall	8.6±2.2	2.1±1.0	5.0±1.1	2.6±1.1
LBBBlarge	8.3±2.2	2.1±0.8	5.1±1.0	2.6±1.0

#### 4.4.4 Influence of image quality

**Fig. 13:**
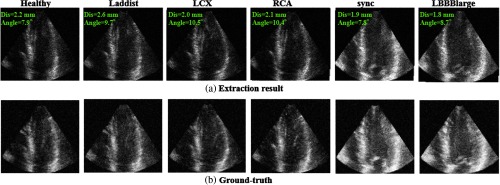
An example of standard plane extraction (synthetic dataset, noise 10%). (a) Extraction result and (b) ground truth.

**Fig. 14:**
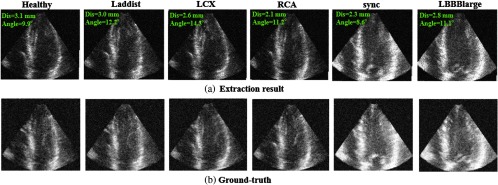
An example of standard plane extraction (synthetic dataset, noise 20%). (a) Extraction result and (b) ground truth.

To evaluate whether the proposed method is robust for a wide range of image qualities, the synthetic data is added with three different noise levels: 0% (original image), 10%, and 20% in relative amplitude. The original data and the generated noise data are put together to train a new classifier. The evaluation result is shown in Table 6. The angle error and distance error slightly increase with increasing noise levels, however, the average error is still under the clinical maximum permissible error. Examples of standard plane extraction under 10% and 20% are shown in Figs. 13 and 14. There is no large difference between the plane extracted from original data and 10% noise. On the other hand, even though the error slightly increases when the noise reaches to 20%, this noise level is likely unrealistically high and would normally not be encountered in clinical recordings. Therefore, this evaluation results suggest that the proposed method is able to deal with a range of image qualities that would be seen in clinical practice.

**Table 6:** Evaluation result on synthetic dataset with a range of image qualities.

	Three long-axis planes		Three short-axis planes	
Noise	Angle (deg)	Distance (mm)	Angle (deg)	Distance (mm)
Original image	8.4±2.2	2.1±0.9	5.1±1.2	2.6±1.2
Noise 10%	9.2±2.8	2.4±1.2	5.9±1.5	2.8±1.3
Noise 20%	11.1±3.3	2.8±1.5	6.6±2.0	3.1±1.5

### 4.5 Discussion

The following factors can be attributed to the improved performance: (1) the proposed method is based on the guideline, where the anatomical regularities are incorporated into determining the initial plane locations. The search regions of each plane were largely cut down. (2) In feature point detection, a coarse-to-fine strategy is proposed for Hough forest classifier, and it also reduces the search region and cuts down noises. (3) The refinement around the initial location using RF further improves the extraction accuracy. The proposed method can be further accelerated by using parallel processing on both Hough forest and RF. Moreover, since the automated method is trained with annotations from multiple observers, it naturally learns a consensus estimation across all the observers and thus less sensitive to bias than manual results.

## 5 Conclusions

This paper proposed a machine learning framework based on the cardiac ultrasound guideline for standard-plane extraction. Each stage in the guideline is achieved using an appropriate machine learning approach. Hough forest with hierarchical search was proposed for detecting efficient and robust feature points. After six planes are extracted by anatomical regularity, a refinement step using RF is applied to improve the accuracy further. Experimental results demonstrate the proposed method is not only fast and accurate, but also robust for a wide range of data, which would be seen in clinical practice.
